# Genomic characterization of a large panel of patient-derived hepatocellular carcinoma xenograft tumor models for preclinical development

**DOI:** 10.18632/oncotarget.3969

**Published:** 2015-05-21

**Authors:** Qingyang Gu, Bin Zhang, Hongye Sun, Qiang Xu, Yexiong Tan, Guan Wang, Qin Luo, Weiguo Xu, Shuqun Yang, Jian Li, Jing Fu, Lei Chen, Shengxian Yuan, Guibai Liang, Qunsheng Ji, Shu-Hui Chen, Chi-Chung Chan, Weiping Zhou, Xiaowei Xu, Hongyang Wang, Douglas D. Fang

**Affiliations:** ^1^ Discovery Services, WuXi AppTec Co., Ltd., Waigaoqiao Free Trade Zone, Shanghai, 200131 China; ^2^ Genome Center, WuXi AppTec Co., Ltd., Waigaoqiao Free Trade Zone, Shanghai, 200131 China; ^3^ Eastern Hepatobiliary Surgery Hospital/Institute of Shanghai, Shanghai, 200131 China; ^4^ Pathology and Laboratory Medicine, Hospital of the University of Pennsylvania, Philadelphia, PA 19104, USA; ^5^ Current address: Cancer Translational Research and In Vivo Pharmacology, China Novartis Institute for Biomedical Research, Zhangjiang Hi-Tech Park, Pudong, Shanghai, 200131 China

**Keywords:** hepatocellular carcinoma (HCC), patient-derived xenograft (PDX) tumor model, copy number alterations (CNA), single nucleotide polymorphism (SNP), fibroblast growth factor receptor (FGFR)

## Abstract

Lack of clinically relevant tumor models dramatically hampers development of effective therapies for hepatocellular carcinoma (HCC). Establishment of patient-derived xenograft (PDX) models that faithfully recapitulate the genetic and phenotypic features of HCC becomes important. In this study, we first established a cohort of 65 stable PDX models of HCC from corresponding Chinese patients. Then we showed that the histology and gene expression patterns of PDX models were highly consistent between xenografts and case-matched original tumors. Genetic alterations, including mutations and DNA copy number alterations (CNAs), of the xenografts correlated well with the published data of HCC patient specimens. Furthermore, differential responses to sorafenib, the standard-of-care agent, in randomly chosen xenografts were unveiled. Finally, in the models expressing high levels of *FGFR1* gene according to the genomic data, FGFR1 inhibitor lenvatinib showed greater efficacy than sorafenib. Taken together, our data indicate that PDX models resemble histopathological and genomic characteristics of clinical HCC tumors, as well as recapitulate the differential responses of HCC patients to the standard-of-care treatment. Overall, this large collection of PDX models becomes a clinically relevant platform for drug screening, biomarker discovery and translational research in preclinical setting.

## INTRODUCTION

Liver cancer is the fifth and seventh most frequently diagnosed cancer in men and women, respectively, worldwide. It is the second and sixth most frequent cause of cancer death in men and women, respectively [1]. Among primary liver cancers, hepatocellular carcinoma (HCC) represents the major histological subtype that accounts for 70% to 85% of the total liver cancers [1]. Surgery is the potentially curative therapy for HCC. Unfortunately, the majority of HCC is unresectable at the time of diagnosis due to advanced disease stages and unfavorable patient conditions [2]. Additionally, HCC is intrinsically resistant to conventional chemotherapies in clinic [3]. Sorafenib, a pan tyrosine kinase inhibitor, has remained the only approved targeted agent for advanced HCC since 2007. Despite of its application in the clinic, the benefit of sorafenib remains modest [4]. The biomarkers predicting the prognosis and responses to the treatment with sorafenib in HCC are virtually absent. Recent studies have shown that a decline in serum AFP levels from baseline may serve as a surrogate marker of outcome during treatment of HCC [5–7]. Development of molecularly targeted agents with novel mechanisms of action is desperately essential to tackle the unmet medical need. However, the success rate of developing new therapies for HCC is low despite exceptional investment in pharmaceutical research and development. Lack of clinically relevant and molecularly characterized preclinical models critically accounts for the failures in the development of efficacious therapeutics for HCC [8].

Establishing patient-derived xenograft (PDX) tumor models in immunodeficient mice has recently generated excitement [9]. PDX models closely resemble the original tumors in the aspects of histopathology, gene-expression patterns, mutational status, and drug responsiveness [9]. In contrast with cancer types prevalent in the Western countries, significantly fewer PDX models for HCC have been developed up to date. The first report of the PDX model for HCC appeared in 1996, in which a metastatic model of HCC from a Chinese patient was established orthotopically in athymic mice [10]. In 2004, also from a Chinese patient, another PDX model was established and reported [11]. In the same year, a group of researchers in Spain reported five HCC PDX models [12]. In 2006, seven subcutaneous PDX models were established from Singapore HCC patients [13]. Obviously, the number of articles that reported PDX models and/or apply PDX models for sequential studies has been increasing gradually. However, these earlier models had not been extensively characterized at the molecular levels, which limited their applications in development of targeted therapy. Furthermore, etiology of HCC appears to be geographically different due to the prevalence of different risk factors. Half of the total HCC-related deaths and new cases occurred in China, which is correlated with a high infection rate of hepatitis B virus (HBV) and hepatitis C virus [3]. Thus, a molecularly understanding of an extensive panel of PDX HCC models derived from Chinese patients is essential for developing more effective therapy.

In this study, we set out to establish a collection of PDX models from Chinese patients. Xenotransplantable grafts were then extensively characterized at histopathological, molecular, and pharmacological levels. We found that PDX tumors retained the histopathological and genomic features of their original counterparts. The landscape of genetic alterations is in agreement with the published data. Differential responses to sorafenib in PDX models reflect the pharmacological heterogeneity observed in the patient population. Efficacy of a novel FGFR1-targeted therapy was further demonstrated in multiple models expressing high levels of *FGFR1* gene. Collectively, molecularly characterized HCC PDX models enable ‘personalized trials’ in mice by selecting potential responders and assist in identification of predictive biomarkers for patient stratification. Such an extensive collection of PDX models will accelerate new target discovery, test of novel therapeutics, and translation of experimental therapies into the clinic.

## MATERIALS AND METHODS

### PDX establishment

**Figure 1:**
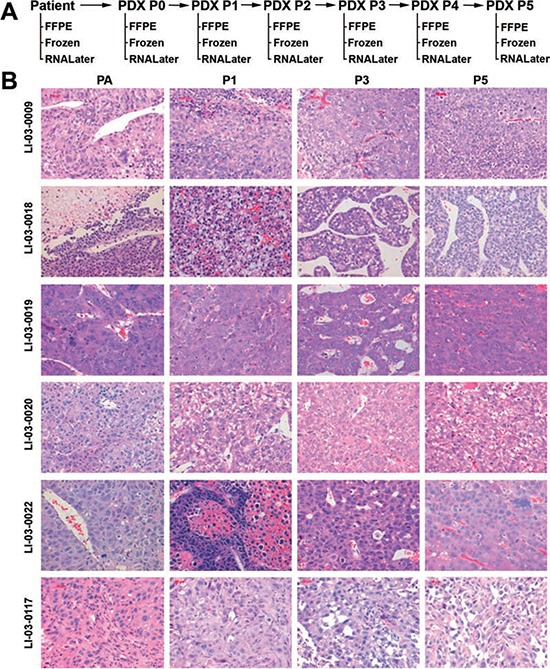
A. Schema depicts the work flow of establishment of PDX models for HCC, including the disposition of patient samples, and PDX tissues at each passage **B.** Representative H&E sections (400 ×) of the original patient tumors and xenografts. PA, patient tumor; P0, the first xenograft in mice; P1, the second xenograft; and beyound.

In compliance with the protocol approved by the Institutional Review Board of Eastern Hepatobiliary Surgery Hospital/Institute of Shanghai and with the subject's informed consent, a fragment of surgically resected tumor tissue was used for xenotransplantation [14]. Briefly, patient samples (designated as PA) were collected, trimmed, cut into 20–30 mm^3^ fragments and implanted subcutaneously in the fore and/or hind bilateral flanks of anesthetized 6- to 8-week old female BALB/c athymic or severe combined immunodeficiency (SCID) mice (Shanghai SLAC Laboratory Animal Co., Ltd.; Shanghai Sino-British Sippr/BK Lab Animal Co., Ltd., Shanghai) within three hours. The mice were examined periodically for three months. Once the first generation of xenografts (named as P0) was established, serial implantations in BALB/c athymic mice were performed to expand the xenograft tumors (i.e. P1, P2, P3, and beyond; Figure 1A). Tumor size was measured using a digital caliper (Cal Pro, Sylvac, Switzerland). Tumor volume was calculated as 0.5 × length × width^2^. Tumor fragments (~200 mm^3^) at each passage were viably frozen in a freezing solution (10% DMSO, 20% FBS, and 70% RPMI 1640 medium) and stored in liquid nitrogen for future re-implantation. Additional fragments were either snap-frozen in liquid nitrogen, or preserved in RNAlater RNA stabilization reagent (Qiagen), or fixed for histology. All procedures and protocols were approved by the Institutional Animal Care and Use Committee of WuXi AppTec.

### Histology

Patient samples and PDX tissues were formalin-fixed, paraffin-embedded, cut into sections, and stained with hematoxylin and eosin (H&E). Histopathology was examined under light microscopy by a pathologist (XX).

### Tissue processing for genomic studies

Genomic DNA and RNA were isolated using a QIAamp DNA mini kit (Qiagen) and RNeasy protect mini kit (Qiagen), respectively. The concentrations were quantified using NanoDrop ND-1000 spectrophotometer (NanoDrop, Wilmington, DE). RNA samples with an RNA integrity number above 8.0 and A_260/280_ ratios above 2.0 were used for gene expression array. DNA samples with A_260/280_ ratios between 1.8 and 2.0 and A_260/230_ ratios above 2.0, and proven to be high quality by gel electrophoresis were used for WES and SNP 6.0 array analyses.

### Gene expression array

Total RNA was amplified and fragmented using a GeneChip® 3′ IVT expression kit (Affymetrix, Santa Clara, CA). Then the samples were hybridized onto a GeneChip® PrimeView™ human gene expression array (Affymetrix). Arrays were scanned on an Affymetrix GeneChip® scanner 3000 7G (Affymetrix). Resulting data was subject to bioinformatics analysis. Briefly, the raw CEL data were processed on an Expression Console™ (version 1.1, Affymetrix). Signal intensities were normalized by the robust multiarray average normalization approach. On 9 pairs of samples which consisted of original patient samples and their corresponding xenograft tumors, unsupervised hierarchical clustering analysis was performed by ‘hclust’ package on R with criteria Euclidian distance and average linkage.

### Whole exome sequencing (WES)

One microgram of each DNA sample was used for library construction using a TruSeq DNA sample preparation kit (Illumina, San Diego, CA). Libraries were pool (500 ng each) for exome capture and amplification using the TruSeq Exome Enrichment kit (Illumina). Sequencing was then performed with paired-end 2 × 100 base reads on the Illumina HiSeq 2000 platform (Illumina). Raw FASTQ files were first processed by a proprietary algorithm to filter out mouse sequence contaminations. We have shown that this filter step does not affect the human SNP detection [15, 16]. After mouse sequence removed, data was aligned to human reference genome hg19/GRCh37 by BWA 0.6.1 and processed to variants calling by GATK 1.6.

### Copy number analysis

Genomic DNA was amplified and fragmented using a core SNP 6 reagent kit and DNase I (Affymetrix). Then the sample is hybridized onto Affymetrix GeneChip® genome-wide human SNP array 6.0 arrays and the arrays were scanned on an Affymetrix GeneChip® scanner 3000 7G. Data was initially analyzed on the Affymetrix Genotyping Console™ and subject to further in-house analysis. Raw CEL data of SNP 6.0 arrays was processed on the Genotyping Console (version 4.1.1.834, Affymetrix). Segment summary of each sample was generated by the Genotyping Console with default configuration. Then all summaries were processed by a proprietary algorithm to retrieve HUGO gene symbols of genes that are located within the segments regions, with copy number status and gene annotations.

### Serum AFP expression

Sera were collected from mice bearing tumors with a total volume of between 300 and 1,000 mm^3^. Fifteen μl of sera was subjected to AFP detection by using an AimPlex multiplex AFP assay kit following the manufacturer's instruction (QuantoBio, Beijing, China). Briefly, AFP specific antibody coated bead suspension was added to a 96-well filter plate. Test sera (15 μl) were added and incubated. Extensive washing was performed afterwards and between each step below. Biotinylated AFP specific antibody (25 μl) was added and incubated. Streptavidin-PE solution (25 μl) was added and incubated. Reading buffer (150 μl) was added to resuspend the beads, and the suspension was subject to flow cytometry analysis using a Canto II (BD Biosciences, San Jose, CA).

### Compounds and therapeutic assays

Sorafenib (LC Laboratories, Woburn, MA) was formulated in CremophorEL/95% ethanol (50:50; Sigma) at 4 × concentration and was diluted with distilled water. Lenvatinib was synthesized by Wuxi AppTec (Shanghai, China) with a purity of 96% and formulated in distilled water. For efficacy studies, female BALB/c athymic mice were implanted with tumor fragments subcutaneously. When the tumors reached 150 – 200 mm^3^, the mice were stratified into different groups for treatments. Each group contained 5 animals. Sorafenib (60 mg/kg), or lenvatinib (30 mg/kg), or vehicles were administered by oral gavage daily. Tumor sizes and body weights were measured twice weekly. Tumor growth inhibition (TGI) is calculated for each group using the formula: TGI (%) = [1−(Ti−T0)/(Vi−V0)] × 100; Ti is the average tumor volume of a treatment group on a given day, T0 is the average tumor volume of the treatment group on the first day of treatment, Vi is the average tumor volume of the vehicle control group on the same day with Ti, and V0 is the average tumor volume of the vehicle group on the first day of treatment. The differences of tumor volumes between groups were analyzed for significance using t test for studies with two groups; or one-way ANOVA followed by Tukey's test for studies with more than two groups.

## RESULTS

### Establishment of PDX models and clinical characteristics of patients

Out of 254 HCC clinical sample implants, we were able to establish 65 xenograft tumors grown in immunodeficient mice as transplantable PDX models (~26%). PDXs were serially passaged in animals 3 – 5 times. The latencies of engraftment and growth rates of PDXs varied significantly among different models (Supplemental Figure 1). A bio-specimen bank of original patient tissues and xenografts at each passages was established, including formalin fixed paraffin-embedded (FFPE) blocks, viably cryopreserved, and RNAlater®-treated and/or snap frozen tissues (Figure 1A). Cryopreserved xenografts could re-grow in mice, providing a renewable tissue source for future experimentations.

Clinical characteristics of these 65 original tumors, including HBV viral infection history of patients tested in the clinic, were summarized in Table 1. Among the collection, ages of patients ranged from 26 up to 78 with medium of 52. Tumor grades ranged from II to IV, and tumor stages from I to IV. All of models were established from treatment-naïve tumors.

**Table 1:** The list of transplantable HCC PDX models, molecular characterizations and clinical information of the corresponding patients

	Model ID	Gender	Age (years)	Source	Histology	Cancer grade	Cancer stage	Metastasis	Hepatitis B virus
1	LI-03-0004	M	63	Primary	HCC	III	T3N0M0	No	+
2	LI-03-0005	M	46	Primary	HCC	III	T3N0M0	No	+
3	LI-03-0006	M	66	Primary	Combined hepatocellular and cholangiocarcinoma	n/a	T2N0M0	No	−
4	LI-03-0007	M	56	Primary	HCC	III	T3N0M1	Yes	−
5	LI-03-0008	M	65	Primary	HCC	III	T2N0M0	No	n/a
6	LI-03-0009	M	53	Primary	HCC	III	T3N0M0	No	+
7	LI-03-0010	M	45	Primary	HCC	III	T3N1M0	No	n/a
8	LI-03-0011	M	37	Primary	HCC	III	T3N0M0	No	+
9	LI-03-0012	M	44	Primary	HCC	III	T3N0M0	No	+
10	LI-03-0014	M	47	Primary	HCC	III	T3N0M0	No	+
11	LI-03-0016	M	42	Primary	HCC	III	T3N0M0	No	+
12	LI-03-0017	F	56	Primary	HCC	III	T3N0M0	No	+
13	LI-03-0018	M	48	Primary	HCC	III	T3N0M0	No	+
14	LI-03-0019	M	46	Primary	HCC	III	T3N0M0	No	+
15	LI-03-0020	M	69	Primary	HCC	III	T3N0M0	No	−
16	LI-03-0021	M	53	Primary	HCC	III	T3N0M0	No	+
17	LI-03-0022	M	46	Primary	HCC	III	T3N0M0	No	+
18	LI-03-0023	F	26	Primary	HCC	III	T3N0M0	No	−
19	LI-03-0055	M	67	Primary	HCC	III	T3N0M0	No	−
20	LI-03-0061	M	57	Primary	HCC	III	T3N0M0	No	+
21	LI-03-0082	M	72	Primary	HCC	III	T3N0M0	No	−
22	LI-03-0086	F	37	Primary	HCC	III	T3N0M0	No	+
23	LI-03-0097	M	55	Primary	HCC	III	T2N0M0	No	−
24	LI-03-0100	F	68	Primary	HCC	III	T3N0M0	No	+
25	LI-03-0101	M	78	Primary	HCC	III	T2N0M0	No	−
26	LI-03-0103	M	58	Primary	HCC	III	T3N0M1	Yes	+
27	LI-03-0113	M	43	Primary	HCC	III	T3N0M0	No	n/a
28	LI-03-0115	M	57	Primary	HCC	III	T2N0M0	No	+
29	LI-03-0117	M	61	Primary	HCC	III–IV	T3N0M0	No	n/a
30	LI-03-0122	F	70	Primary	HCC	III	T3N0M0	No	−
31	LI-03-0126	M	57	Primary	HCC	III	T4N0M0	No	n/a
32	LI-03-0140	F	72	Primary	HCC	III	T1N0M0	No	+
33	LI-03-0141	M	35	Primary	HCC	II	T1N0M0	No	+
34	LI-03-0143	M	47	Primary	HCC	III	T2N0M1	Yes	+
35	LI-03-0146	F	71	Primary	HCC	III	T3N0M0	No	+
36	LI-03-0147	M	53	Primary	HCC	III	T2N0M0	No	−
37	LI-03-0149	M	45	Primary	HCC	III	T1N0M0	No	−
38	LI-03-0153	M	45	Primary	HCC	III	T3N0M0	No	+
39	LI-03-0155	M	46	Primary	HCC	III	T1N0M0	No	+
40	LI-03-0158	F	55	Primary	HCC	III	T4N0M0	No	+
41	LI-03-0159	F	28	Primary	HCC	III	T3N0M0	No	−
42	LI-03-0164	M	42	Primary	HCC	III	T3N0M1	Yes	−
43	LI-03-0167	M	71	Primary	HCC	III	T3N0M0	No	+
44	LI-03-0185	M	64	Primary	HCC	III	T3N0M0	No	+
45	LI-03-0187	M	49	Primary	HCC	III	T1N0M0	No	+
46	LI-03-0189	M	47	Primary	HCC	III	T1N0M0	No	+
47	LI-03-0191	M	34	Primary	HCC	III	T3N1M0	No	+
48	LI-03-0196	M	55	Primary	HCC	III	T2N0M0	No	−
49	LI-03-0198	M	52	Primary	HCC	III	T3N0M0	No	+
50	LI-03-0200	M	44	Primary	HCC	III	T1N0M0	No	+
51	LI-03-0208	M	45	Primary	HCC	III–IV	T3N0M0	No	+
52	LI-03-0209	M	66	Primary	HCC	III	T2N0M0	No	n/a
53	LI-03-0217	M	54	Primary	HCC with sarcomatous change	IV	T4N1M1	Yes	+
54	LI-03-0219	F	33	Primary	HCC	III	T1N0M0	No	+
55	LI-03-0220	M	47	Primary	HCC	III	T3N0M0	No	n/a
56	LI-03-0228	M	43	Primary	HCC	III	T4N0M0	No	+
57	LI-03-0240	M	52	Primary	HCC	III	T1N0M0	No	+
58	LI-03-0242	M	38	Primary	HCC	III	T3N0M0	No	+
59	LI-03-0243	F	46	Primary	HCC	III	T3N0M0	No	+
60	LI-03-0252	M	40	Primary	HCC	III	T3N1M0	No	+
61	LI-03-0254	M	64	Primary	HCC	III	T4N0M0	No	n/a
62	LI-03-0255	M	61	Primary	HCC	III	T2N0M0	No	n/a
63	LI-03-0257	M	53	Primary	HCC	III	T2N0M0	No	+
64	LI-03-0266	M	51	Primary	HCC	III–IV	T1N0M1	Yes	+
65	LI-03-0271	M	36	Primary	HCC	III	T4N0M1	Yes	+

	Model ID	Treatment prior to surgery (chemotherapy or radiotherapy)	Gene expression		Whole exome sequencing (xenograft)	SNP 6.0 array (xenograft)	AFP in sera of tumor-bearing animal (ng/ml)
			Patient, PA, sample	Xenograft			
1	LI-03-0004	None	N/D	P3	P3	P3	N/D
2	LI-03-0005	None	N/D	P3	P2	P2	0
3	LI-03-0006	None	N/D	P3	P3	P3	N/D
4	LI-03-0007	None	N/D	P4	P3	P2	N/D
5	LI-03-0008	None	N/D	N/D	P4	N/D	N/D
6	LI-03-0009	None	N/D	P4	P4	P4	N/D
7	LI-03-0010	None	N/D	P3	P4	P4	0
8	LI-03-0011	None	N/D	P3	P3	P3	N/D
9	LI-03-0012	None	PA	P3	P3	P3	0.01746
10	LI-03-0014	None	N/D	P4	P3	P3	0.01746
11	LI-03-0016	None	N/D	P3	P2	P2	N/D
12	LI-03-0017	None	N/D	P3	P3	P3	N/D
13	LI-03-0018	None	PA	P3	P3	P3	> 820.150
14	LI-03-0019	None	PA	P3	P3	P3	4.645
15	LI-03-0020	None	PA	P3	P3	P3	1.649
16	LI-03-0021	None	PA	P3	P3	P3	N/D
17	LI-03-0022	None	PA	P3	P3	P3	0.307
18	LI-03-0023	None	N/D	N/D	P2	N/D	0.198
19	LI-03-0055	None	N/D	N/D	P2	N/D	N/D
20	LI-03-0061	None	N/D	P3	P2	P2	1.82
21	LI-03-0082	None	N/D	N/D	N/D	N/D	12.225
22	LI-03-0086	None	N/D	P3	P2	P2	121.764
23	LI-03-0097	None	PA	P3	P3	P3	N/D
24	LI-03-0100	None	PA	P3	P3	N/D	N/D
25	LI-03-0101	None	N/D	N/D	P2	N/D	> 820.150
26	LI-03-0103	None	N/D	N/D	N/D	N/D	2.594
27	LI-03-0113	None	N/D	N/D	N/D	N/D	N/D
28	LI-03-0115	None	N/D	P4	P3	P3	> 820.150
29	LI-03-0117	None	N/D	P3	P2	P2	0
30	LI-03-0122	None	N/D	P2	P2	P2	0
31	LI-03-0126	None	N/D	N/D	N/D	N/D	N/D
32	LI-03-0140	None	N/D	P4	P3	P3	> 820.150
33	LI-03-0141	None	PA	P3	P2	P2	N/D
34	LI-03-0143	None	N/D	P3	P3	P3	0.797
35	LI-03-0146	None	N/D	P3	P3	P3	N/D
36	LI-03-0147	None	N/D	P3	P3	P3	N/D
37	LI-03-0149	None	N/D	P2	P2	P2	N/D
38	LI-03-0153	None	N/D	N/D	N/D	N/D	N/D
39	LI-03-0155	None	N/D	N/D	P3	N/D	N/D
40	LI-03-0158	None	N/D	N/D	N/D	N/D	N/D
41	LI-03-0159	None	N/D	P3	P3	P3	> 820.150
42	LI-03-0164	None	N/D	P3	P0	P0	N/D
43	LI-03-0167	None	N/D	N/D	P3	N/D	N/D
44	LI-03-0185	None	N/D	P3	P2	P2	N/D
45	LI-03-0187	None	N/D	P3	P2	P2	N/D
46	LI-03-0189	None	N/D	P3	P2	P2	N/D
47	LI-03-0191	None	N/D	P3	P2	P2	4.11
48	LI-03-0196	None	N/D	N/D	N/D	N/D	820.15
49	LI-03-0198	None	N/D	N/D	P2	N/D	82.104
50	LI-03-0200	None	N/D	P2	P2	P2	N/D
51	LI-03-0208	None	N/D	N/D	P2	N/D	0
52	LI-03-0209	None	N/D	P2	P2	P2	N/D
53	LI-03-0217	None	N/D	P4	P3	P3	0
54	LI-03-0219	None	N/D	N/D	N/D	N/D	16.049
55	LI-03-0220	None	N/D	N/D	P3	N/D	16.169
56	LI-03-0228	None	N/D	N/D	P2	N/D	> 820.150
57	LI-03-0240	None	N/D	P3	P1	P1	N/D
58	LI-03-0242	None	N/D	N/D	P3	N/D	N/D
59	LI-03-0243	None	N/D	P3	P2	P2	146.279
60	LI-03-0252	None	N/D	P3	P2ww	P2	N/D
61	LI-03-0254	None	N/D	P3	P2	P2	79.602
62	LI-03-0255	None	N/D	N/D	P3	N/D	N/D
63	LI-03-0257	None	N/D	N/D	P3	N/D	N/D
64	LI-03-0266	None	N/D	N/D	N/D	N/D	0.553
65	LI-03-0271	None	N/D	P4	P2	P2	148.491
			9	43	56	42	

Note: N/D, not done; n/a, not available; P, passage; AFP, serum-fetoprotein (AFP).

### Histology of xenografts

The histology and degree of differentiation of the PDX matched well with that of the original tumor (Figure 1B). In addition, PDX tumors maintained intratumor heterogeneity, resembling the original tumors (Figure 1B).

### Molecular characterization of xenografts and/or corresponding patients' tumors

We then determined gene expression profiles, mutational status, CNAs, and serum AFP expression in PDX models by expression array, WES, SNP 6.0 array, and flow cytometry, respectively.

### Gene expression

**Figure 2:**
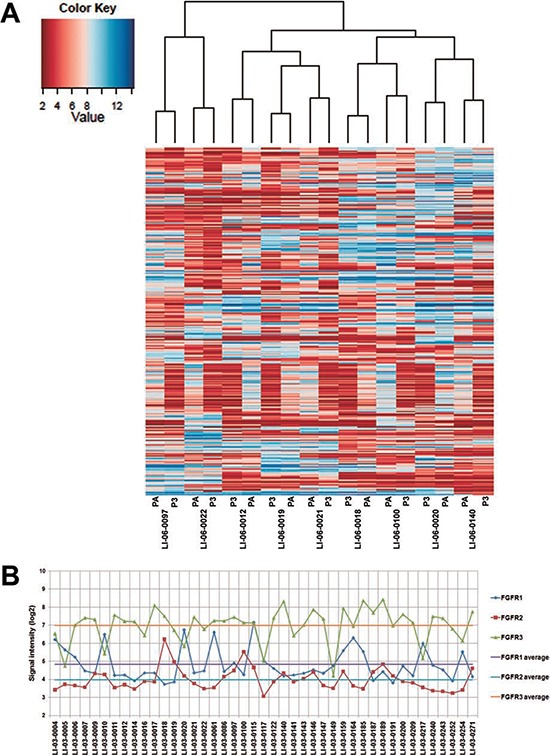
Gene expression profiles in the original tumors were well maintained in PDXs **A.** The dendrogram shows unsupervised hierarchical clustering of samples according to gene expression pattern, and heat map after substraction of genes whose intensity value's standard deviation is < 1.8 across all samples is shown. **B.** Expression levels, represented by signal intensities, of FGFR1, FGFR2, and FGFR3 in 44 models. The average of expression levels of each gene was calculated and marked as color-coded solid lines.

In order to establish a correlation of gene expression profiles between the original and xenograft tumors, we first performed a pilot study in randomly selected nine models indicated in Figure 2A. Each model comprised of the original patient tumors and corresponding xenograft tumors (P3). Unsupervised clustering of the results showed that all of nice PDXs clustered tightly together with the corresponding patient tumors, demonstrating that PDXs authentically maintained the global gene expression characteristics of patients' tumors (Figure 2A).

To further identify the set of genes which discriminated the PDXs from the original tumors, we analyzed the differentially expressed genes between two populations. The results showed that, compared to the original tumors, 83 probe sets revealed the upregulation of approximately 72 genes in xenografts with an average fold change of 3.5, whereas 749 probe sets detected the downregulation of approximately 744 genes in PDX tumors (Supplemental Table 1).

Gene expression profiling was further performed in xenografts of additional 34 models. All expression data from 9 patient tumors and 43 PDXs have been deposited to Gene Expression Omnibus (http://www.ncbi.nlm.nih.gov/geo/; accession GSE55828). Thus, the global gene expression signature in 43 models has been established, which provides a database for searching for PDX models exhibiting an appropriate biomarker for targeted therapy. Notably, overexpression of *FGFR1* gene was observed in several models, which may discriminate a population sensitive to *FGFR1*-targeted agents (Figure 2B). Three models, designated as LI-03-0010, LI-03-0020 and LI-03-0164, which overexpressed *FGFR1* but not *FGFR2* or *FGFR3* genes in comparison with the average levels of the collection of models, were selected for subsequent *in vivo* pharmacological studies.

### WES

WES was performed to detect mutational alterations, including SNPs and indels, in protein-coding regions in 56 xenograft tumors at a coverage of 100 ×. Xenografts at P2 or P3 from 51 models were used with a few exceptions (Table 1).

We first found that mouse DNA sequences accounted for a significant fraction of sequence reads in xenograft samples, even using a human exome capture kit. In order to remove murine DNA sequences from the xenograft sequencing data, we have established a proprietary algorithm to deplete mouse sequence contamination and keep human sequence reads intact. The performance of this algorithm has been demonstrated in a previous study of exome sequencing of eight case-matched trio sets of Chinese esophageal squamous cell carcinoma, blood, and PDX tumor tissues [15, 16]. An example was elucidated in Supplemental Figure 2A. In average, murine DNA reads contributed to approximately 40% of genetic variations detected in PDX tissues. Excluding these contaminating mouse sequences significantly improved the accuracy of mutation detection in xenograft samples.

Across all 56 PDX models, we detected an average of 8033.5 protein-altering single nucleotide alterations (SNA) per model, including 86 frame-shift indels, 3.5 stop-gain/loss indels, 7878 non-synonymous SNPs, and 66 stop-gain/loss SNPs. A complete list of genetic alterations in 56 models was provided in Supplemental Table 2.

A variety of genetic alterations causing amino-acid changes (i.e., non-synonymous alterations, including SNPs and indels) on the genes have previously been linked to HCC [17–19]. In our study, we identified many of them in the 56 models (Supplemental Table 2). Regarding *TP53*, genetic alterations were found in 42 models (75%), including a variety of SNPs and 2 deletions. Twelve models carried 13 genetic alterations in *MLL3* gene, including 7 frame shift insertions and 6 SNPs (21%). For *CTNNB1*, we found SNPs in 8 models (14%). Seven models showed SNPs in *AXIN1* gene and another exhibited deletion (14%). Five models showed distinct SNPs in *ARID1B* gene (9%). Four models carried SNP, or deletion, or insertion of *RB1* gene (7%). Four models carried distinct SNPs of *JAK1* gene (7%). Three exhibited SNPs of *TP73* gene (5%). Two models carried a SNP and a deletion, respectively, in *CDKN2A* gene (4%). One model carried a SNP in *KRAS* gene (2%). One model carried a SNP in *IGF2R* (2%).

### Copy number analysis

We performed genome-wide human SNP 6.0 arrays to determine DNA CNA across the entire human genome in 42 PDX tumors. The results were summarised in Supplemental Table 3. Genes exhibited normal copy numbers (i.e., 2) were left blank. Otherwise, 3 or 4, or higher numbers indicated a copy number gain and 0 or 1 indicated a copy number loss. A comparison of CNA with published data [20] was illustrated in Table 2. For example, copy number losses of *AFF1* (76%), *RAP1GDS1* (71%), *TP53* (64%), *PTEN* (19%) genes were identified. Copy number gains were detected in *PBX1* (76%), *PRCC* (76%), *ARNT* (62%), *MYC* (62%), *BCL9* (60%), *MTDH* (52%), *MET* (43%), and *FGF19* (14%).

**Table 2:** Comparison of representative amplified and deleted cancer genes between 286 HCC patient samples reported by Wang et al. (16) and our 42 HCC PDX models

Rank	CNA type	Gene	Frequency in patients % (Illumina Human Omin1_Quad BeadChip)	Frequency in PDX models % (Affymetric SNP 6.0 array)
1	Amplification	PBX1	N/A	76.2
2	Amplification	PRCC	N/A	76.2
3	Amplification	ARNT	12.9	61.9
4	Amplification	BCL9	8.7	59.5
5	Amplification	MTDH	12.9	52.4
6	Amplification	COX6C	12.6	52.4
7	Amplification	ABL2	12.9	50.0
8	Amplification	MET	4.5	42.9
9	Amplification	CCND1	4.9	16.7
10	Amplification	FGF19	4.9	14.3
1	Deletion	AFF1	19.6	76.2
2	Deletion	RAP1GDS1	19.6	71.4
3	Deletion	WRN	15.7	71.4
4	Deletion	PCM1	17.1	71.4
5	Deletion	WHSC1L1	17.1	66.7
6	Deletion	RB1	9.1	59.5
7	Deletion	BRCA2	7.0	57.1
8	Deletion	CDKN2A	12.6	57.1
9	Deletion	CDH1	N/A	50.0
10	Deletion	CDKN2B	12.6	45.2
11	Deletion	TSC2	N/A	38.1
12	Deletion	SMAD4	4.9	33.3
13	Deletion	APC	N/A	28.6
14	Deletion	STK11	N/A	26.2
15	Deletion	WT1	N/A	23.8
16	Deletion	MLH1	N/A	21.4
17	Deletion	TNFAIP3	6.3	21.4
18	Deletion	PTEN	4.9	19.1
19	Deletion	CDKN2C	7.0	16.7
20	Deletion	ARID1A	7.0	14.3
21	Deletion	TNFRSF14	8.0	11.9

Note: N/A, information not available.

### Detection of AFP in serum in PDX models

Among 32 models tested, serum AFP was detectable in 26 models (81.3%; Table 1), suggesting that a large fraction of PDX models reflect the characteristics of HCC in clinic.

### Differential responses to sorafenib and efficacy of FGFR inhibitor lenvatinib

**Figure 3:**
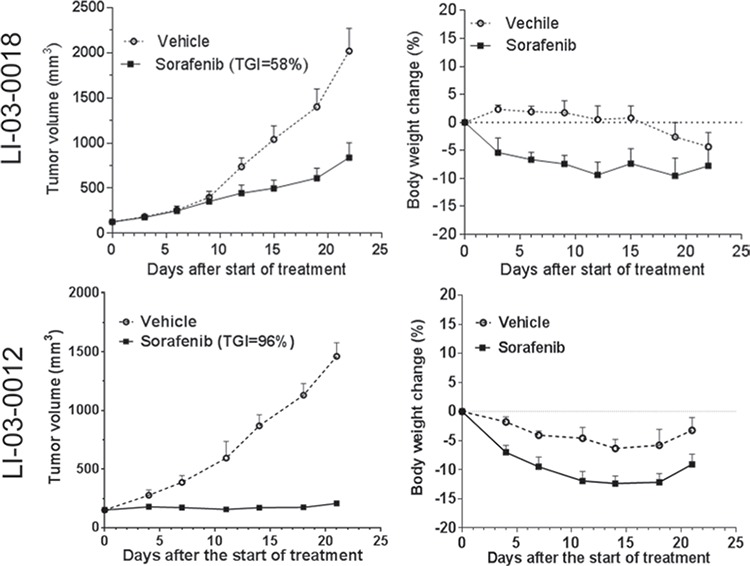
The effect of standard-of-care compound sorafenib was evaluated in two HCC models (LI-03-0018 at P6; LI-03-0012 at P5) Tumor-bearing animals were treated for 22 and 21 days in LI-03-0018 and LI-03-0012 models, respectively. Tumor volumes (left panel) and body weight changes (right panel) were plotted by the mean ± standard error mean. At the end point, compared to vehicle controls, tumor growth inhibition induced by the treatment with sorafenib was statistically significant with variable confidence P levels: *P* < 0.05 and *P* < 0.01 in LI-03-0018 and LI-03-0012 models, respectively. No statistical difference in the changes of body weights was identified between vehicle and treatment groups in both experiments (*P* > 0.05).

To evaluate the responses of PDX models to the standard-of-care agent, we first conducted *in vivo* efficacy studies of sorafenib in two randomly selected PDX models (Figure 3). Interestingly, one model, designated as LI-03-0018, showed a partial response (TGI, 58%) whereas another LI-03-0012 showed a stable disease (TGI, 96%). These results suggest that, similar to the heterogeneous patient population in clinic, PDX models responded to the standard-of-care therapy differentially.

**Figure 4:**
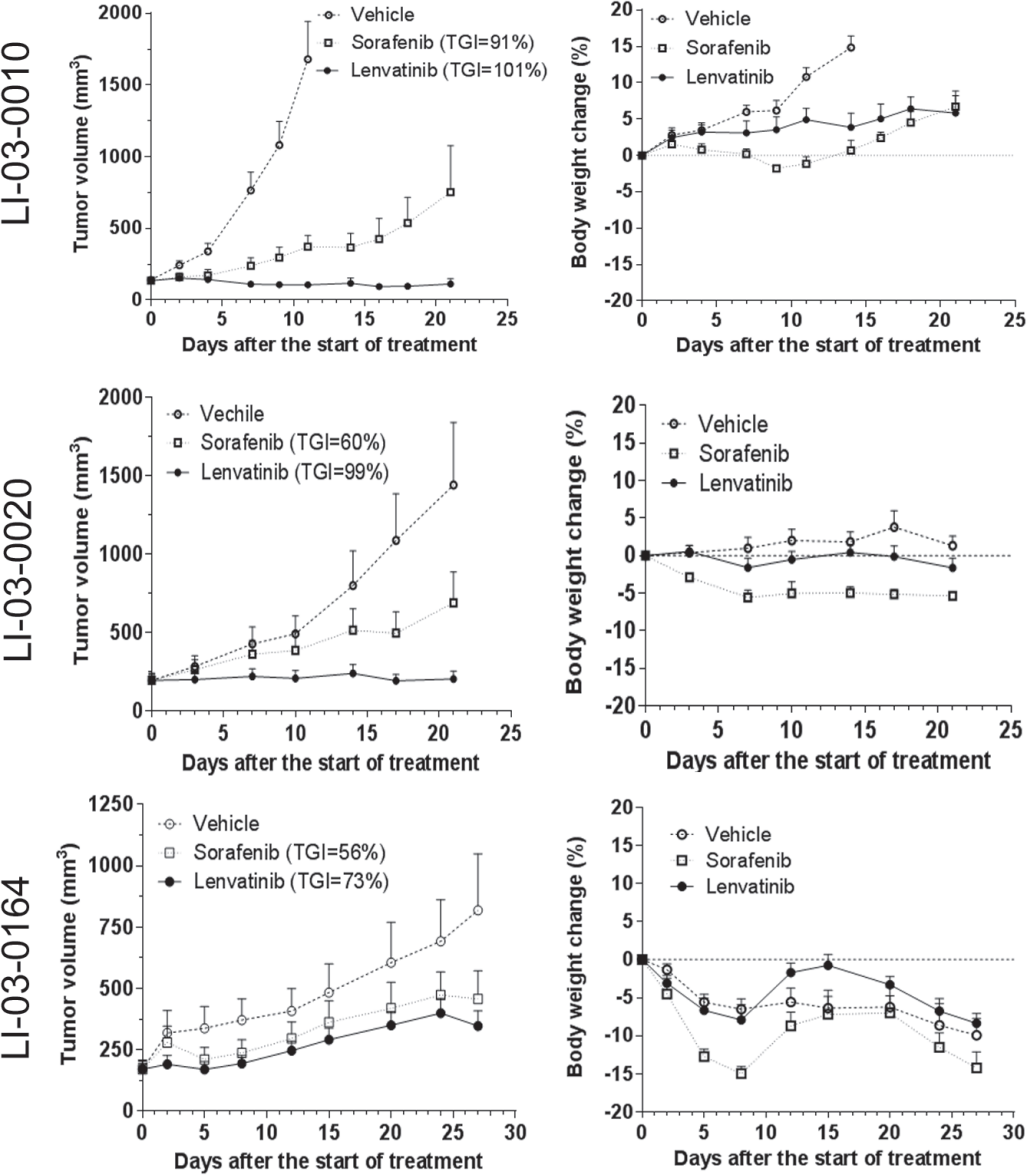
The effects of FGFR inhibitor lenvatinib and sorafenib were evaluated in *FGFR1*-overexpressing models LI-0010 (P8), LI-0020 (P6), and LI-03-0164 (P5) In the study in LI-03-0010, tumor-bearing animals were treated for 21 days with lenvatinib or sorafenib, whereas the vehicle group was terminated at day 11 due to its rapid growth. Statistical analyses were performed using the data at d11. In comparison with the tumor sizes in the control group, significant tumor growth inhibition was observed in both sorafenib- (*P* < 0.05) and lenvatinib-treated (*P* < 0.05) groups. No significant difference was observed between two agents (*P* > 0.05). Loss of body weight was observed in both treatment groups when compared with the control group (*P* < 0.05) and, additionally, a significant difference existed between two treatment groups (*P* < 0.05). In the study in LI-03-0020, all of tumor-bearing animals in there groups were treated for 21 days. At the end point, significant growth inhibition was only observed in lenvatinib-treated group compared to the control group (*P* < 0.05). however, a significant loss of body weight only appeared in sorafenib-treated group compared to the control group (*P* < 0.05). In the study in LI-03-0164, tumor-bearing animals were treated for 22 days. No significant difference in tumor growth inhibition or body weight was observed in this study (*P* > 0.05).

In order to elucidate the application of the molecularly characterized PDX models in personalized medicine, we selected three models LI-03-0010, LI-03-0020 and LI-03-0164 with overexpression of *FGFR1* gene, but with lower-than-average levels of *FGFR2/3* genes (Figure 2B), for targeted therapy of FGFR inhibitor lenvatinib. Our results showed that *FGFR1*-overexpressing models were more sensitive to the treatment with lenvatinib in comparison with sorafenib (Figure 4). TGIs of lenvatinib and sorafenib were 101% and 91%, respectively, in the model of LI-03-0010; 99% and 60% in LI-03-0020; and 73% and 56% in LI-03-0164. Although no statistically significant growth inhibition was observed between lenvatinib and sorafenib treatment groups, a greater effect of lenvatinib was clearly demonstrated, especially in the model of LI-03-0020, in which statistically significant tumor growth inhibition was only induced by the treatment with lenvanitib, but not with sorafenib, in comparison with vehicle control.

In all three *in vivo* experiments, body weights of lenvatinib-treated mice were maintained better than sorafenib-treated mice (Figure 4). A profound weight loss (i.e., ≥ 20%) only appeared in sorafenib-treated group in LI-03-0164, which led to treatment discontinuation since d8. These results implicate for less toxicity of lenvatinib compared to sorafenib.

## DISCUSSION

In this study, we have established a large collection of serially transplantable PDX models for HCC. The collection of PDX models recapitulates the features of the original tumors, including histopathology, gene expression profiles, mutational status, DNA CNA, and a serum biomarker, making it an excellent tool for study of HCC and drug discovery.

An earlier study showed that the differences of gene expression between xenografts and original patient samples were relatively low through the passages up to P9 [21]. In the present study, we characterized gene expression profiles of xenografts collected at P3, at which passage the tumor materials usually become sufficient to perform robust efficacy studies. The results demonstrate that xenografts accurately reflect the global expression profiles of the original tumors with limited changes. In agreement with earlier studies of colorectal cancer [21], among the genes down-regulated in xenografts with respect to patient tumors, we observed enrichment in genes encoding for extracellular matrix components, cell adhesion molecules, and immune system regulators (Supplemental Table 1). The data suggest that modification of gene expression in xenografts is attributable to the loss of the human stromal components and the infiltration of mouse stromal cells. Conversely, genes associated with cell cycle and DNA replication were up-regulated in PDX tissues, suggesting that the enrichment of the tumorigenic cell population. Overall, despite of the limited differences caused by the distinct tumor microenvironment, PDX tissues authentically retain the gene expression patterns of the original tumors.

It is noteworthy that enhancer of zeste homolog 2 (EZH2) is one of 72 genes upregulated in xenografts. EZH2 plays an important role in HCC tumorigenesis [22] and its up-regulation was associated with HCC progression and metastasis [23, 24]. Moreover, EZH2 regulates the self-renewal and differentiation of murine hepatic stem/progenitor cells [25] and tumor-initiating HCC cells are highly dependent on EZH2 for their tumorigenic activity [26]. It has been shown that EZH2 concordantly silences the Wnt pathway antagonists operating at several subcellular compartments, which in turn activate Wnt/β-catenin signaling in HCC, and concomitant overexpression of EZH2 and β-catenin was observed in one-third of HCC cases and significantly correlated with tumor progression [23]. Furthermore, Short-hairpin RNA and pharmacological inhibition of EZH2 impaired HCC cell growth and anchorage-independent sphere formation of HCC cells in culture [26]. Knockdown of EZH2 in HCC cell lines suppressed HCC motility *in vitro* and pulmonary metastasis in an athymic mouse model [24]. Collectively, considering of the enrichment of tumorigenic cancer cells at xenotransplanation, our findings agree that pharmacological interference with EZH2 might be a promising therapeutic approach to targeting HCC.

Mutations in various genes have been reported in primary HCC specimen, including *TP53, CTNNB1, AXIN1, RB1, TP73, CDKN2A, KRAS* and *IGF2R* [17–19]. In agreement with the literature, WES analyses revealed that these genes were frequently mutated in 56 PDX models with an incidence of 75%, 14%, 14%, 7%, 5%, 4%, 2%, and 2%, respectively. Therefore, our data obtained from PDX models confirm the potential linkage of these genes with HCC development and progression.

In addition to identifying known mutations in HCC, a number of novel SNPs and indels were uncovered through WES of PDX samples. Most notably, phosphodiesterase 4D interacting protein (*PDE4DIP*) gene ranks on the top of genetic alterations (i.e., SNPs and indels; Supplemental Table 2), showing 190 genetic alterations in all of 56 models tested (100%). These alterations include 1 insertion, 19 deletions, and 170 SNPs. The protein encoded by *PDE4DIP* serves to anchor phosphodiesterase 4D to the Golgi/centrosome region of the cell. *PDE4DIP* was reported to fuse to *PDGRFB* gene in myeloproliferative disorders [27]. The expression of serum PDE4DIP protein was linked to esophageal squamous cell carcinoma [28]. The potential role of *PDE4DIP* in HCC tumorigenesis requires further investigation. Moreover, alterations of *SARM1* gene were frequently observed in PDX models with 111 SNPs and 1 deletion in 55 models (98%). *SARM1* is a negative regulator of MYD88- and TRIF-dependent toll-like receptor signalling pathway involved in innate immune response. Genetic alterations of this gene were frequently observed in esophageal adenocarcinoma [29], colon and rectal cancer [30], and lung adenocarcinoma [31]. These novel alterations identified in PDX models warrant further studies to explore their biological functions in HCC tumorigenesis. Overall, the spectrum of protein-altering genetic variations in our PDX collection is similar to that reported in the literature for HCC. PDX tumor grafts retain the molecular features of original tumors.

The genomic landscape of HCC, especially CNA, has been extensively characterized in 286 paired tumor and adjacent non-tumor tissues [20]. This work led to identification of 29 amplification peaks and 22 deletion peaks with high confidence [20]. Notably, our overall CNA pattern is consistent with the earlier study (Table 2), indicating that the PDX models recapitulate the genetic CNA landscape of HCC clinical samples. For instance, the oncogenic role of *BCL9* and *MTDH* genes has been established in the recent study [20]. *BCL9* encodes B-cell CLL/lymphoma 9, which is involved in the WNT/β-catenin signaling pathway by mediating the recruitment of pypopus to the nuclear β-catenin-TCF complex [32]. An oncogenic role of *MTDH* gene, which encodes metadherin, has been implicated in a variety of cancer types including HCC [33]. Gene expression and protein expression (assessed by immunohistochemistry) of BCL9 and MTDH correlated with their somatic copy numbers [20]. Inhibition of *BCL9* and *MTDH* expression mediated by siRNA significantly suppressed cell proliferation of HCC cell lines with *BCL9* and *MTDH* gene amplifications, respectively, but not in copy number neutral cell lines [20]. Consistent to the earlier report, the copy number gains of both genes were identified in 60% and 52% of PDX models, respectively, in our study. The frequencies of amplification of both genes ranked on the top of the gene lists identified in PDX models (Table 2), which is in agreement with that both *BCL9* and *MTDH* genes may play an important role in HCC development. In addition, the potential oncogenic role of *PRDM14* and *FRWD2* genes has been implicated in earlier report [20]. We also found that amplifications of *PRDM14* and *RFWD2* gene in 41% and 57%, respectively, of PDX models (Supplemental Table 3). Our results support that both genes are worthy the further investigation as oncogenic drivers of HCC.

AFP has been considered a biomarker for prognosis and treatment outcome [5–7]. In this study, similar to clinical patients, a large fraction of HCC models exhibited the elevated levels of serum AFP, demonstrating that PDX models may serve as an *in vivo* system for evaluating serum AFP in the preclinical setting.

The promise of PDX models is to accelerate the development of novel therapeutics. PDXs derived from HCC patient samples have been utilized for *in vivo* pharmacological tests of of several drugs, including anti-VEGF antibody bevaciumab [34], mTOR inhibitors sirolimus [35] and everolimus [36], dual inhibitor of VEGFR and FGFR brivanib [37], sorafenib [35, 38, 39], EGFR inhibitor gefitinib [13], MEK1/2 inhibitor AZD6244 [39, 40], VEGFR and PDGFR inhibitor sunitinib [38, 41], VEGFR-2 and C-MET inhibitor foretinib [42], FGFR, VEGFR and PDGFR inhibitor dovitinib [43]. In the present study, we first showed the differential responses to the treatment with sorafenib in two randomly selected models, indicating that the panel of models represents the distinct outcomes of a diverse HCC patient population in clinic.

Developing potential predictive markers to identify the responders in the patient population is the key for the success of clinical development. Molecular characterized PDX models are an excellent *in vivo* system to explore predicative biomarkers for various targeted agents. As an example, in *FGFR1*-overexpressing models, LI-03-0010, LI-03-0020, and LI-03-0164, we demonstrated that lenvatinib exhibited antitumor activity greater than sorafenib. Additionally, the treatment with lenvatinib appeared to be better tolerated in animals when compared with sorafenib because the first agent never caused dosing suspension while the latter did that in the model of LI-03-0164. In the tested models, the elevated levels of *FGFR1* gene, but not *FGFR2* or *FGFR3* genes, were detected by expression assays regarding to their basal levels in a panel of PDX models (Figure 2B), suggesting that *FGFR1* expression levels may be further investigated as a predictive biomarker for the therapy of lenvatinib.

Lenvatinib was very recently approved by US FDA for the treatment of patients with locally recurrent or metastics, progressive, radioactive iodine-refreactory differentiated thyroid cancer [44]. Lenvatinib is a non-selective tyrosine kinase inhibitor [45]. It selectively targets FGFR1 among FGFR1-4. However, as a multikinase inhibitor, lenvatinib also inhibits VEGFR1-3, PDGFR-α and PDGFR-β [45]. Different from sorafenib, it was speculated that the effects of lenvatinib on thyroid cancer could be mediated by the inhibition of unique targets of lenvantinib, including FGFR1 [44]. Further studies are warranted to elucidate the mechanism of action for tumor growth inhibition in *FGFR1*-overexpressing HCC PDX models, and to confirm the genomic correlation of the drug sensitivity with overexpression of *FGFR1* gene. Clearly, our results demonstrated that such a sub-population of HCC patients may respond to the treatment with lenvatinib better than sorafenib. Overall, our data provide evidence that FGF/FGFR pathway is a therapeutic target for the treatment of a subpopulation of HCC patients. Especially, as lenvatinib is recently approved by FDA for the treatment of patients with thyroid cancer, a rapid clinical development of the drug in HCC may become feasible.

In conclusion, we have developed an extensive panel of PDX models for HCC, which authentically maintain the histopathological and molecular characteristics of the original tumors. Such a panel of molecularly characterized animal models for HCC provides an excellent opportunity to study the biology of HCC, to develop novel therapy, as well as to facilitate research of personalized medicine.

## SUPPLEMENTARY FIGURES AND TABLES








