# Supplementary material for: Genomic characterization of a large panel of patient-derived hepatocellular carcinoma xenograft tumor models for preclinical development
Source: Oncotarget. 2015 May 21;6(24):20160–76. doi: 10.18632/oncotarget.3969 (PMC4652995; doi:10.18632/oncotarget.3969)
Supplement: Supplementary file 1 [file oncotarget-06-20160-s001.pdf]

SUPPLEMENTARY FIGURES AND TABLES

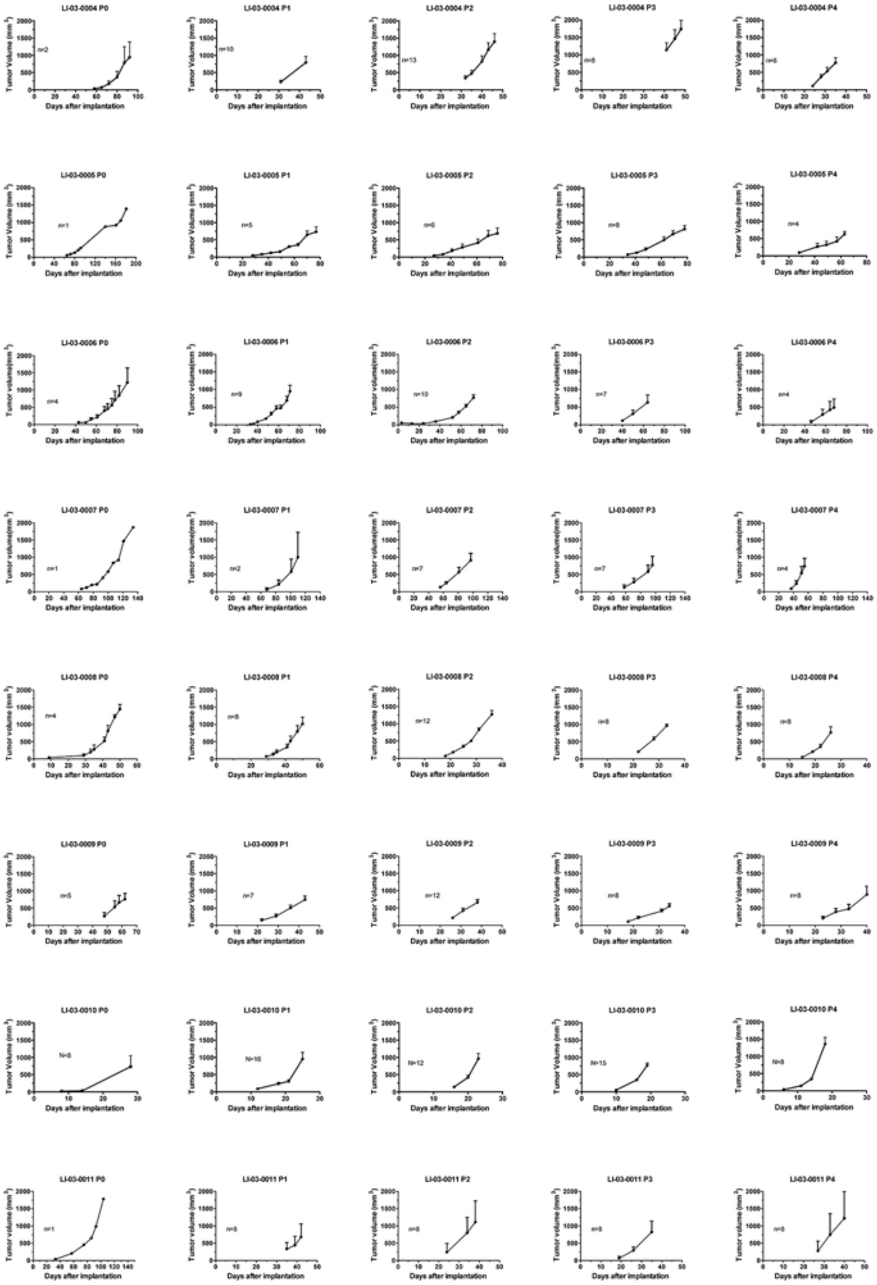

(*Continued*)

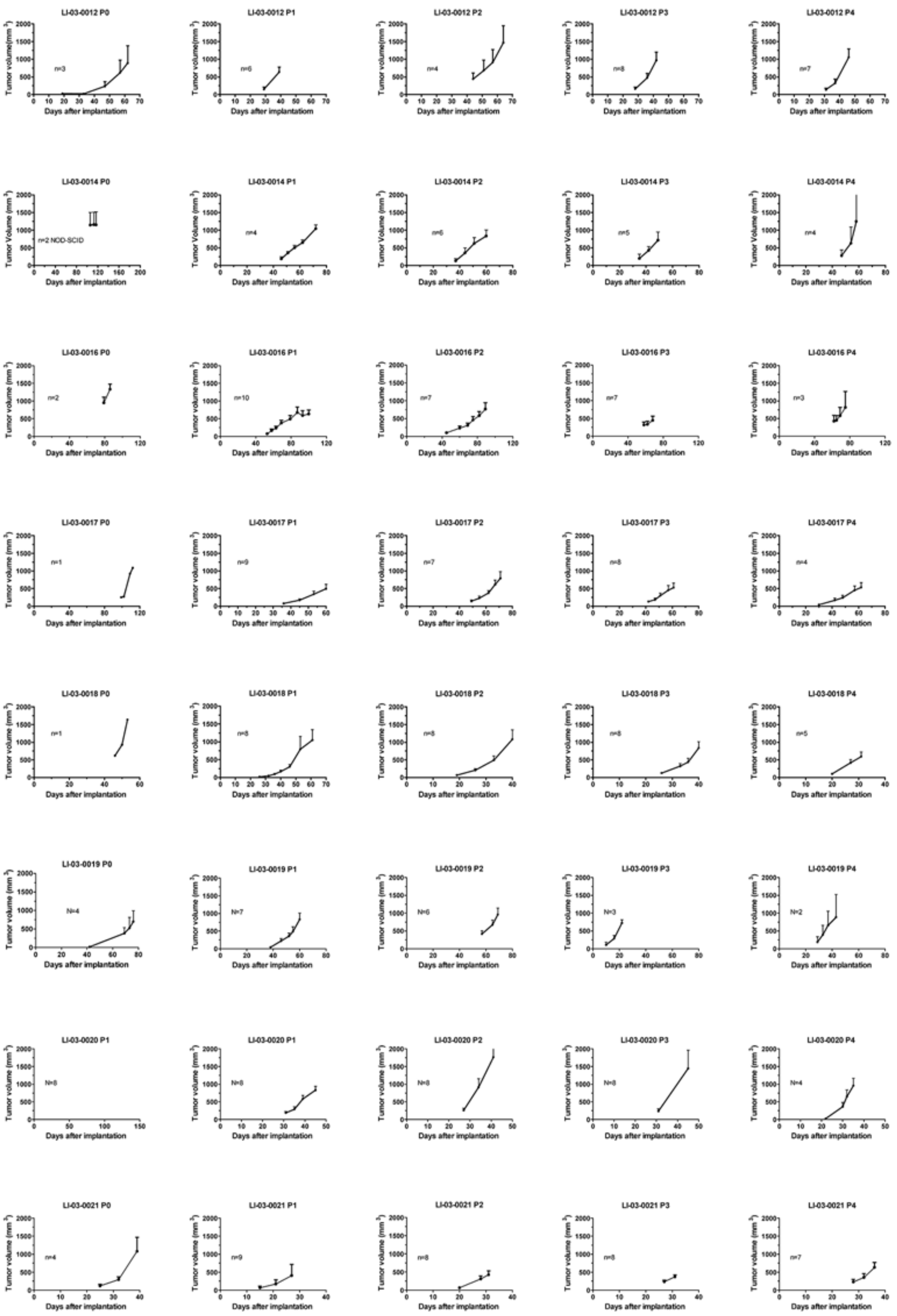
LI-03-0012 P0
LI-03-0012 P1
LI-03-0012 P2
LI-03-0012 P3
LI-03-0012 P4
LI-03-0014 P0
LI-03-0014 P1
LI-03-0014 P2
LI-03-0014 P3
LI-03-0014 P4
LI-03-0016 P0
LI-03-0016 P1
LI-03-0016 P2
LI-03-0016 P3
LI-03-0016 P4
LI-03-0017 P0
LI-03-0017 P1
LI-03-0017 P2
LI-03-0017 P3
LI-03-0017 P4
LI-03-0018 P0
LI-03-0018 P1
LI-03-0018 P2
LI-03-0018 P3
LI-03-0018 P4
LI-03-0019 P0
LI-03-0019 P1
LI-03-0019 P2
LI-03-0019 P3
LI-03-0019 P4
LI-03-0020 P1
LI-03-0020 P1
LI-03-0020 P2
LI-03-0020 P3
LI-03-0020 P4
LI-03-0021 P0
LI-03-0021 P1
LI-03-0021 P2
LI-03-0021 P3
LI-03-0021 P4
Tumor volume (mm³)
Days after implantation

(Continued)

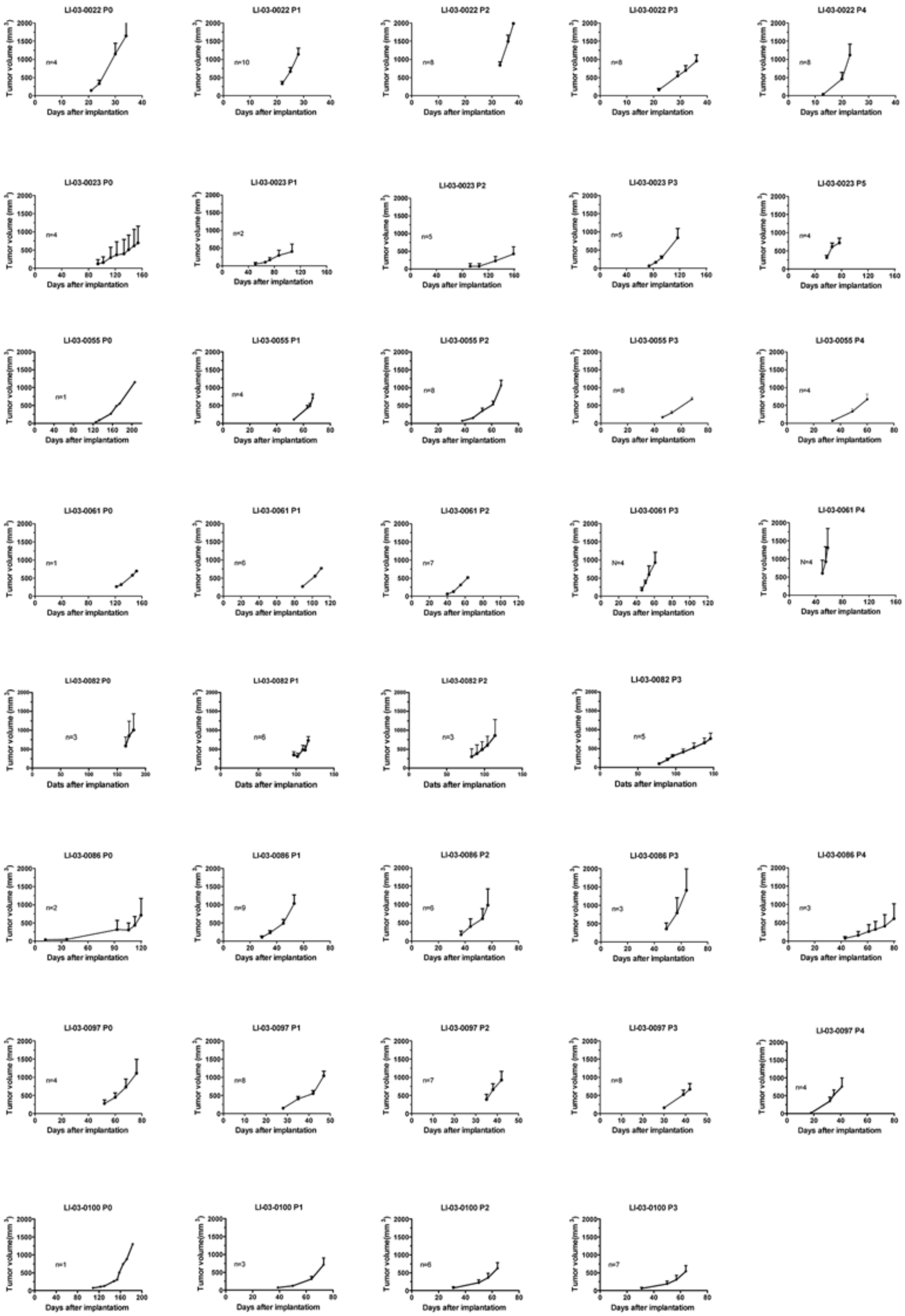
LI-03-0022 P0
LI-03-0022 P1
LI-03-0022 P2
LI-03-0022 P3
LI-03-0022 P4
LI-03-0023 P0
LI-03-0023 P1
LI-03-0023 P2
LI-03-0023 P3
LI-03-0023 P5
LI-03-0055 P0
LI-03-0055 P1
LI-03-0055 P2
LI-03-0055 P3
LI-03-0055 P4
LI-03-0061 P0
LI-03-0061 P1
LI-03-0061 P2
LI-03-0061 P3
LI-03-0061 P4
LI-03-0082 P0
LI-03-0082 P1
LI-03-0082 P2
LI-03-0082 P3
LI-03-0086 P0
LI-03-0086 P1
LI-03-0086 P2
LI-03-0086 P3
LI-03-0086 P4
LI-03-0097 P0
LI-03-0097 P1
LI-03-0097 P2
LI-03-0097 P3
LI-03-0097 P4
LI-03-0100 P0
LI-03-0100 P1
LI-03-0100 P2
LI-03-0100 P3

(*Continued*)

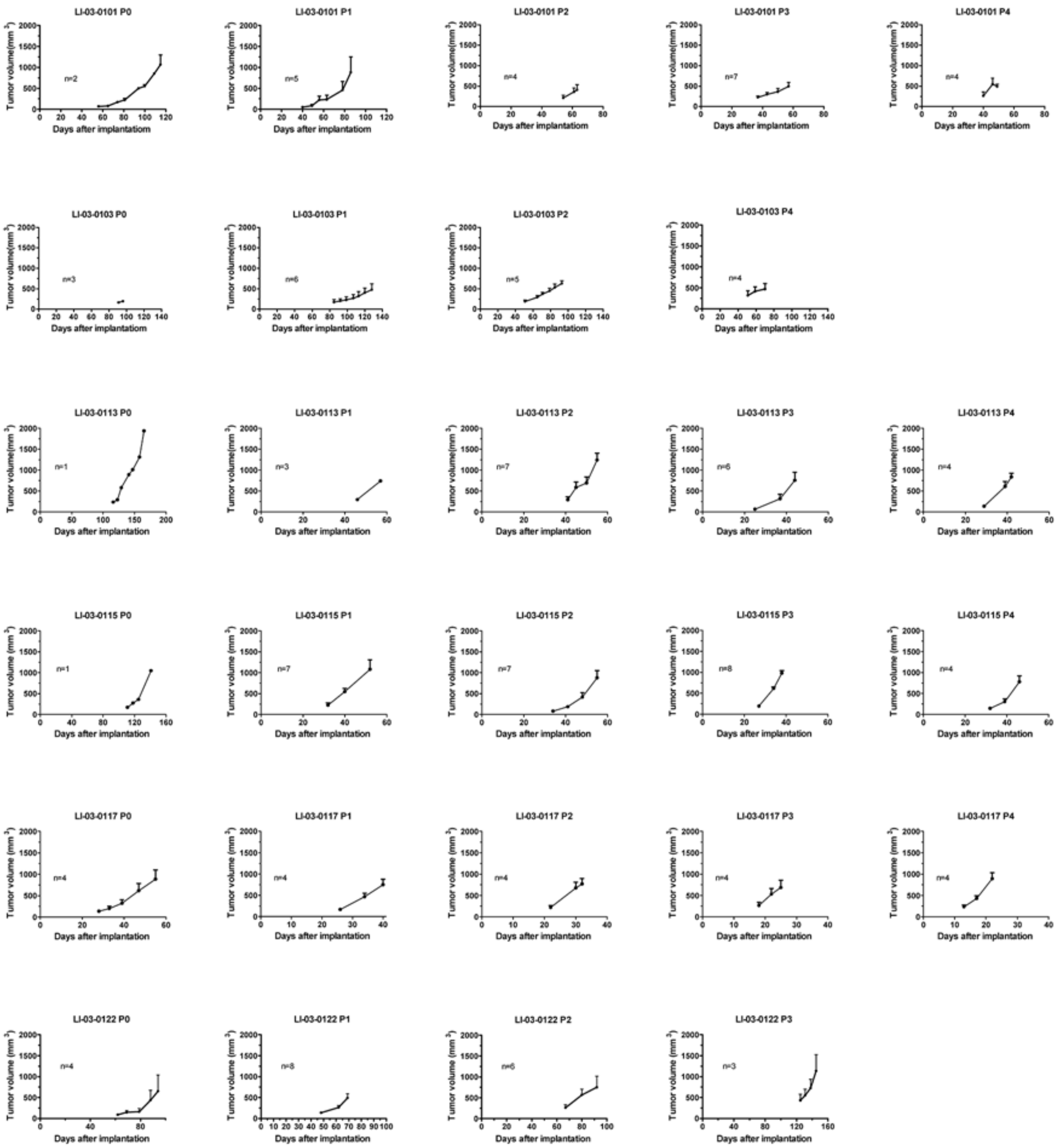
LI-03-0101 P0
n=2
LI-03-0101 P1
n=5
LI-03-0101 P2
n=4
LI-03-0101 P3
n=7
LI-03-0101 P4
n=4
LI-03-0103 P0
n=3
LI-03-0103 P1
n=6
LI-03-0103 P2
n=5
LI-03-0103 P4
n=4
LI-03-0113 P0
n=1
LI-03-0113 P1
n=3
LI-03-0113 P2
n=7
LI-03-0113 P3
n=6
LI-03-0113 P4
n=4
LI-03-0115 P0
n=1
LI-03-0115 P1
n=7
LI-03-0115 P2
n=7
LI-03-0115 P3
n=8
LI-03-0115 P4
n=4
LI-03-0117 P0
n=4
LI-03-0117 P1
n=4
LI-03-0117 P2
n=4
LI-03-0117 P3
n=4
LI-03-0117 P4
n=4
LI-03-0122 P0
n=4
LI-03-0122 P1
n=8
LI-03-0122 P2
n=6
LI-03-0122 P3
n=3
Tumor volume(mm 3)
Days after implantation

(*Continued*)

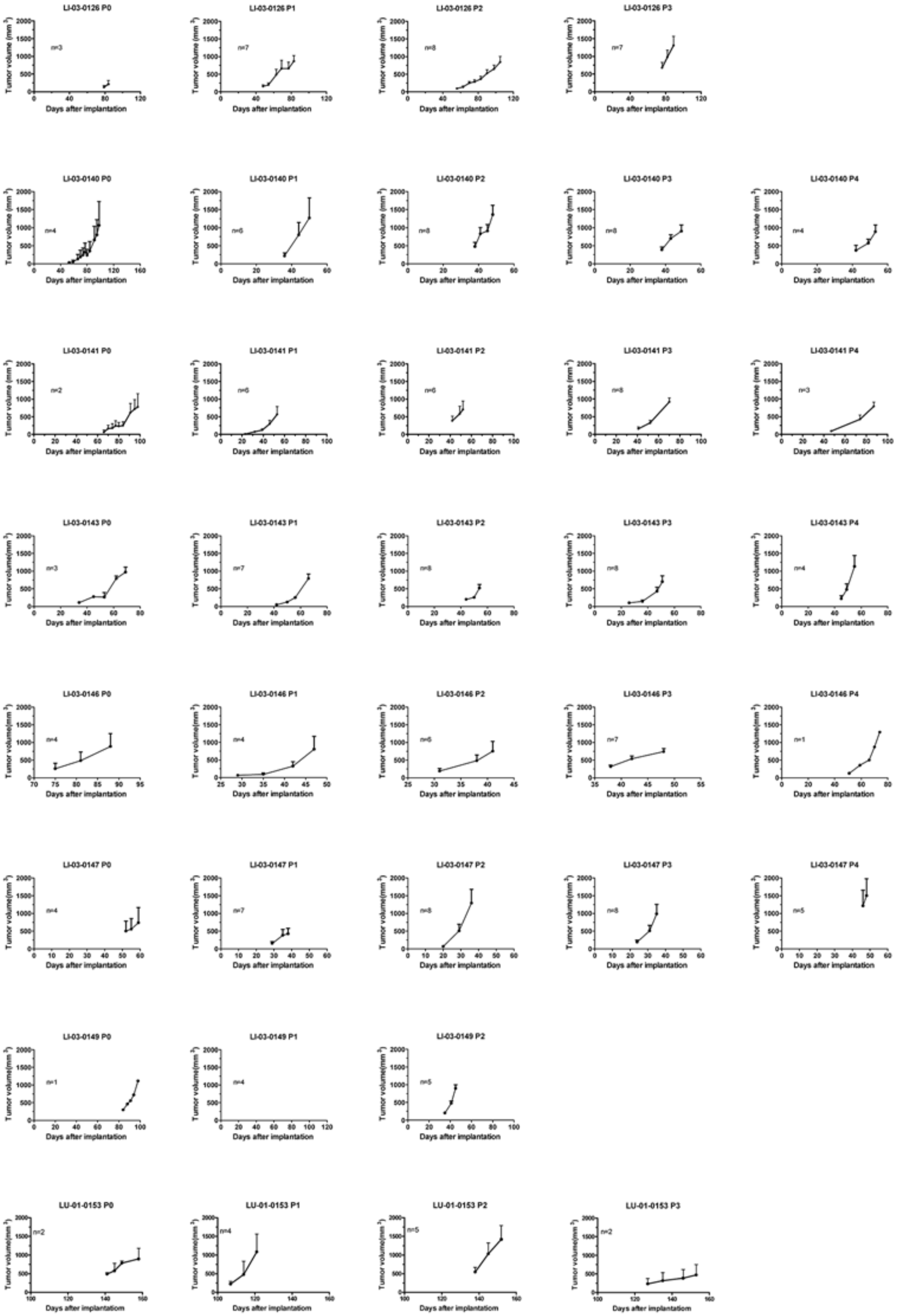
LI-03-0126 P0
LI-03-0126 P1
LI-03-0126 P2
LI-03-0126 P3
LI-03-0140 P0
LI-03-0140 P1
LI-03-0140 P2
LI-03-0140 P3
LI-03-0140 P4
LI-03-0141 P0
LI-03-0141 P1
LI-03-0141 P2
LI-03-0141 P3
LI-03-0141 P4
LI-03-0143 P0
LI-03-0143 P1
LI-03-0143 P2
LI-03-0143 P3
LI-03-0143 P4
LI-03-0146 P0
LI-03-0146 P1
LI-03-0146 P2
LI-03-0146 P3
LI-03-0146 P4
LI-03-0147 P0
LI-03-0147 P1
LI-03-0147 P2
LI-03-0147 P3
LI-03-0147 P4
LI-03-0149 P0
LI-03-0149 P1
LI-03-0149 P2
LU-01-0153 P0
LU-01-0153 P1
LU-01-0153 P2
LU-01-0153 P3
Tumor volume (mm 3)
Days after implantation

(*Continued*)

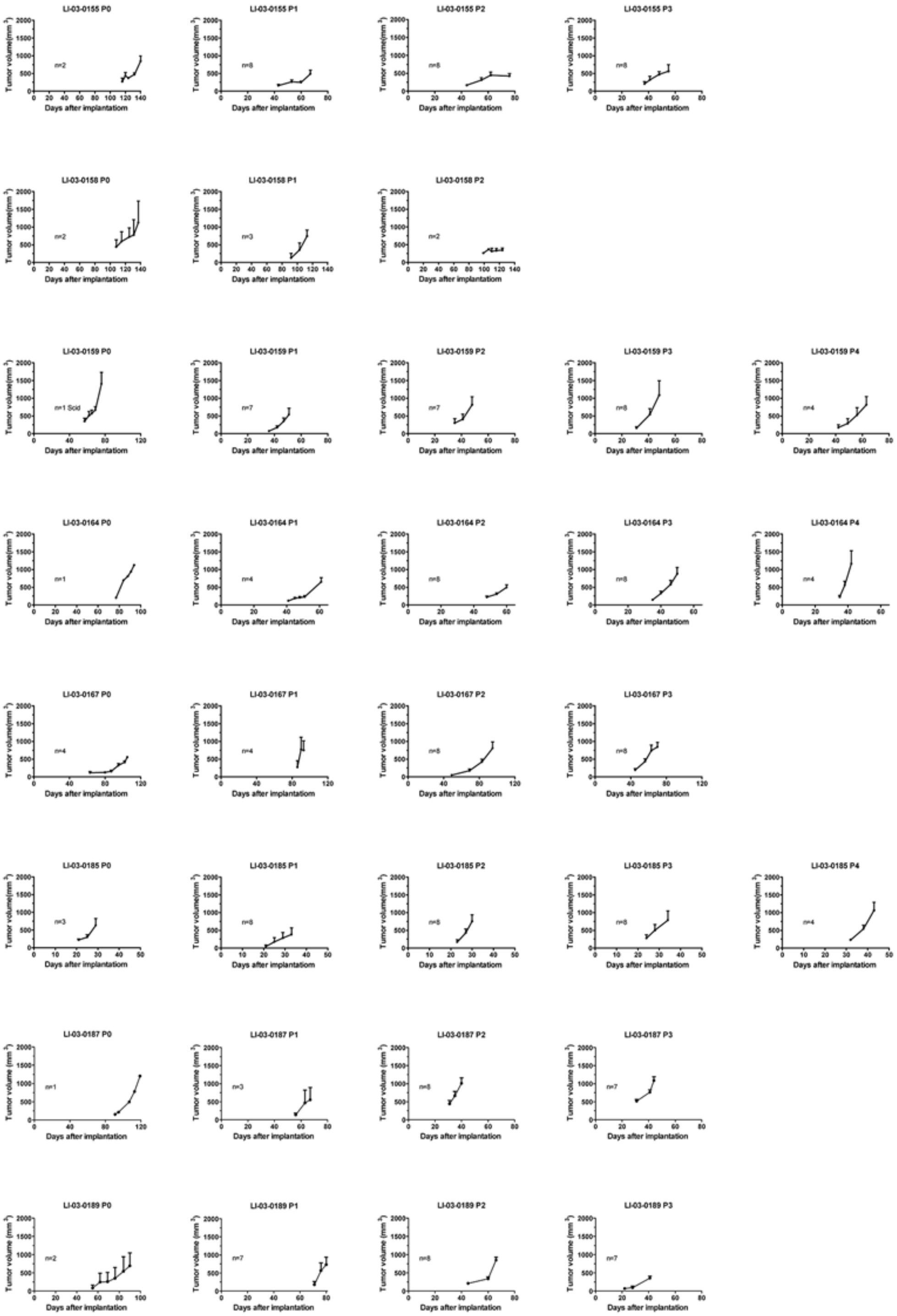
LI-03-0155 P0
LI-03-0155 P1
LI-03-0155 P2
LI-03-0155 P3
LI-03-0158 P0
LI-03-0158 P1
LI-03-0158 P2
LI-03-0159 P0
LI-03-0159 P1
LI-03-0159 P2
LI-03-0159 P3
LI-03-0159 P4
LI-03-0164 P0
LI-03-0164 P1
LI-03-0164 P2
LI-03-0164 P3
LI-03-0164 P4
LI-03-0167 P0
LI-03-0167 P1
LI-03-0167 P2
LI-03-0167 P3
LI-03-0185 P0
LI-03-0185 P1
LI-03-0185 P2
LI-03-0185 P3
LI-03-0185 P4
LI-03-0187 P0
LI-03-0187 P1
LI-03-0187 P2
LI-03-0187 P3
LI-03-0189 P0
LI-03-0189 P1
LI-03-0189 P2
LI-03-0189 P3

(*Continued*)

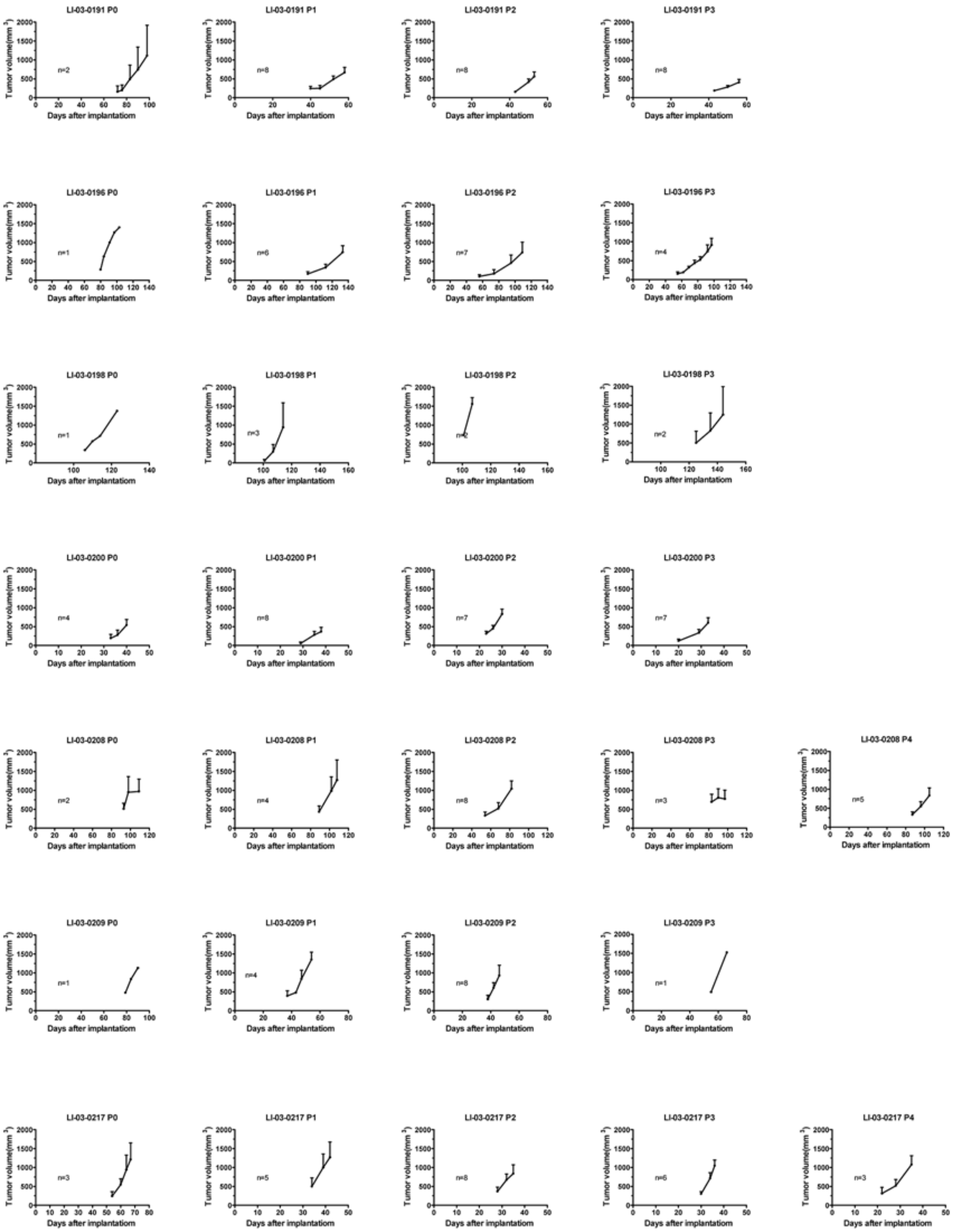
LI-03-0191 P0
n=2
LI-03-0191 P1
n=8
LI-03-0191 P2
n=8
LI-03-0191 P3
n=8
LI-03-0196 P0
n=1
LI-03-0196 P1
n=6
LI-03-0196 P2
n=7
LI-03-0196 P3
n=4
LI-03-0198 P0
n=1
LI-03-0198 P1
n=3
LI-03-0198 P2
n=2
LI-03-0198 P3
n=2
LI-03-0200 P0
n=4
LI-03-0200 P1
n=8
LI-03-0200 P2
n=7
LI-03-0200 P3
n=7
LI-03-0208 P0
n=2
LI-03-0208 P1
n=4
LI-03-0208 P2
n=8
LI-03-0208 P3
n=3
LI-03-0208 P4
n=5
LI-03-0209 P0
n=1
LI-03-0209 P1
n=4
LI-03-0209 P2
n=8
LI-03-0209 P3
n=1
LI-03-0217 P0
n=3
LI-03-0217 P1
n=5
LI-03-0217 P2
n=8
LI-03-0217 P3
n=6
LI-03-0217 P4
n=3
Tumor volume(mm 3)
Days after implantatiom

(*Continued*)

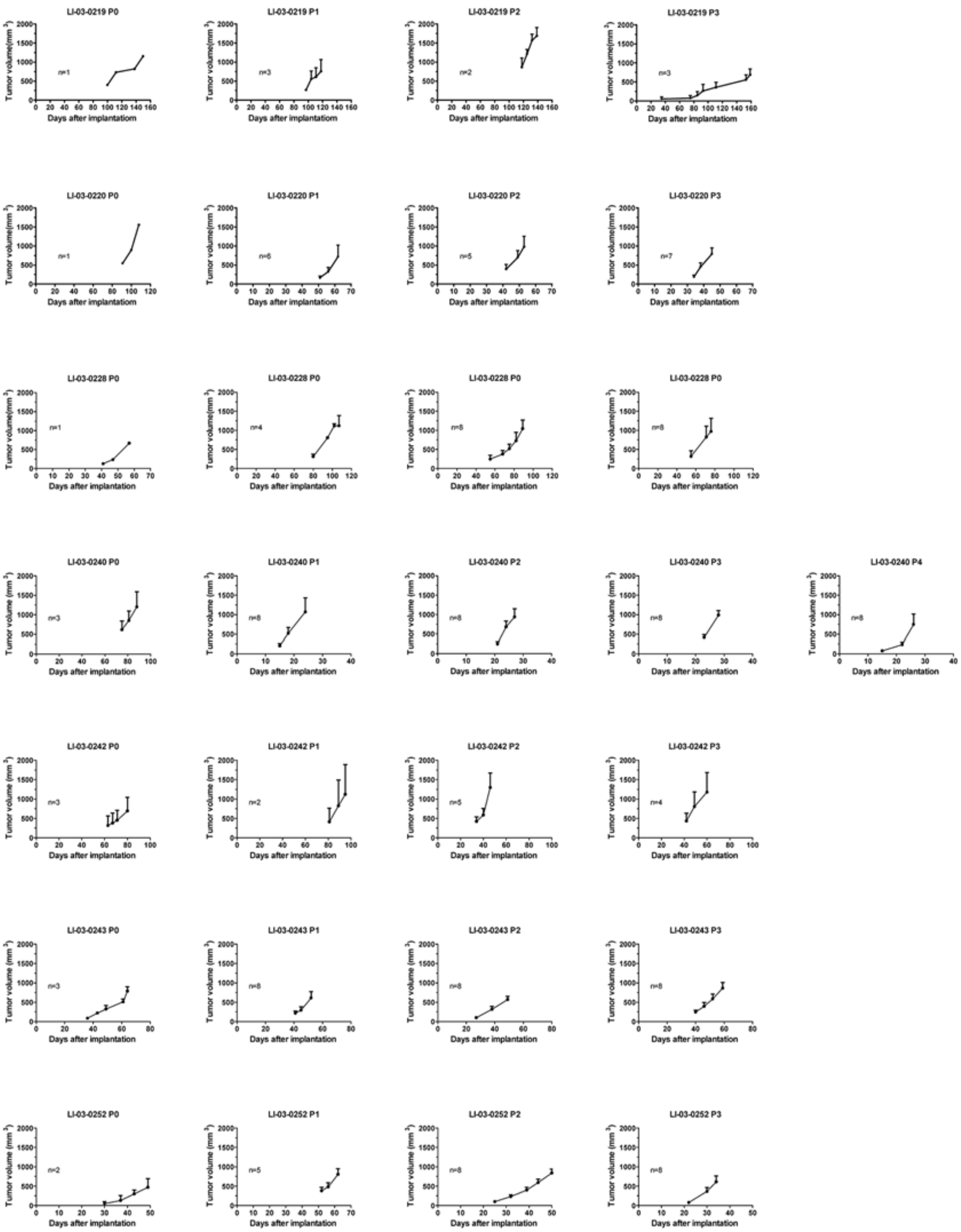
LI-03-0219 P0
n=1
LI-03-0219 P1
n=3
LI-03-0219 P2
n=2
LI-03-0219 P3
n=3
LI-03-0220 P0
n=1
LI-03-0220 P1
n=6
LI-03-0220 P2
n=5
LI-03-0220 P3
n=7
LI-03-0228 P0
n=1
LI-03-0228 P0
n=4
LI-03-0228 P0
n=8
LI-03-0228 P0
n=8
LI-03-0240 P0
n=3
LI-03-0240 P1
n=8
LI-03-0240 P2
n=8
LI-03-0240 P3
n=8
LI-03-0240 P4
n=8
LI-03-0242 P0
n=3
LI-03-0242 P1
n=2
LI-03-0242 P2
n=5
LI-03-0242 P3
n=4
LI-03-0243 P0
n=3
LI-03-0243 P1
n=8
LI-03-0243 P2
n=8
LI-03-0243 P3
n=8
LI-03-0252 P0
n=2
LI-03-0252 P1
n=5
LI-03-0252 P2
n=8
LI-03-0252 P3
n=8
Tumor volume(mm 3)
Tumor volume (mm 3)
Days after implantatiom
Days after implantation

(*Continued*)

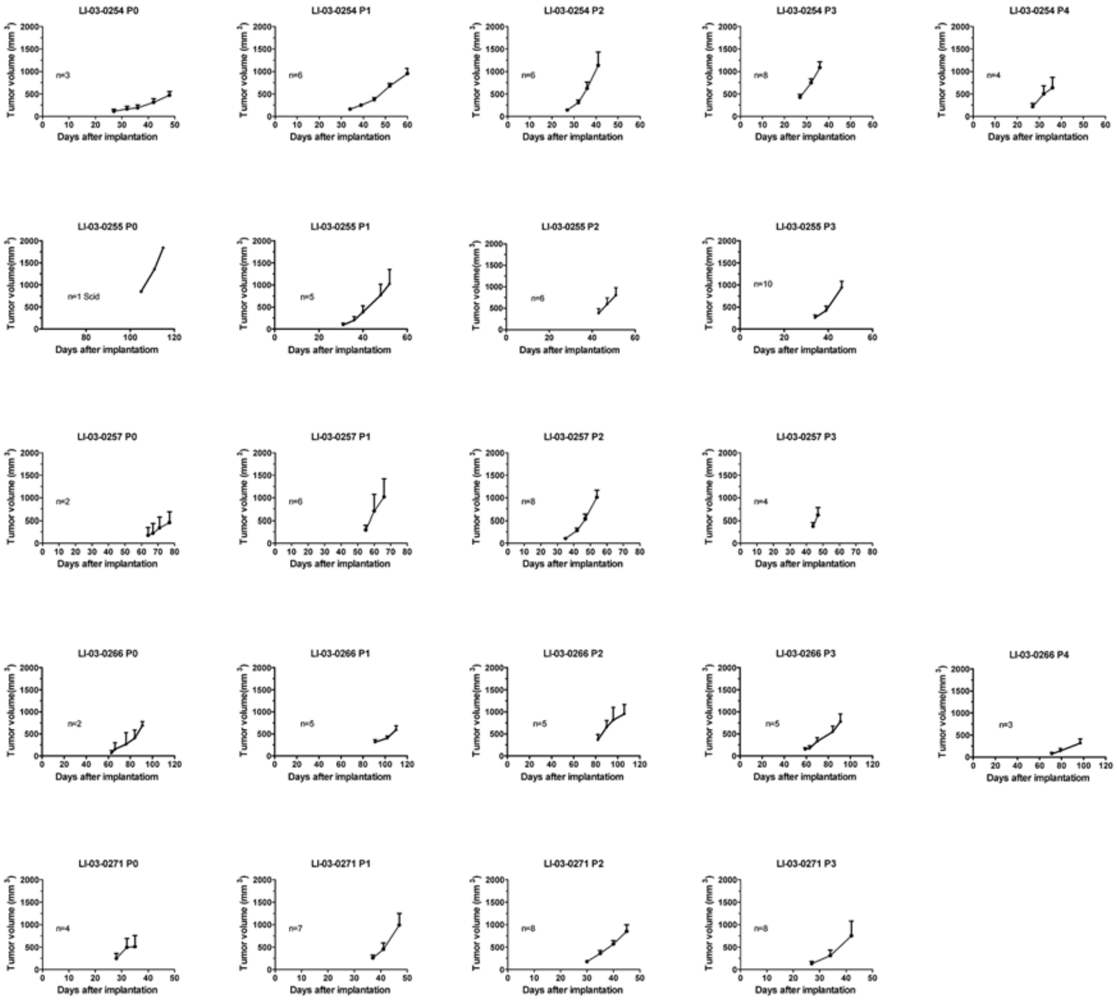

Supplementary Figure S1: The growth curves of 65 transplantable PDX models at various passages in the present study. The data shown are mean ± standard error mean (SEM).

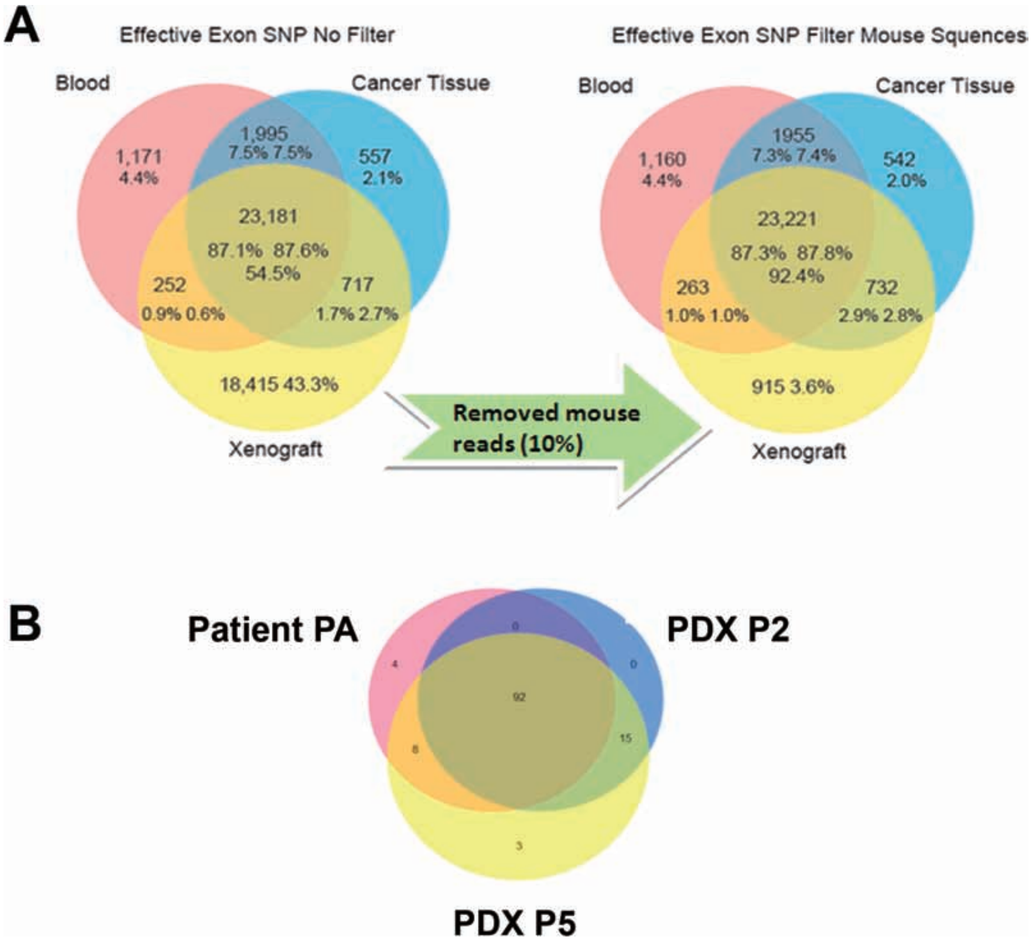

Supplementary Figure S2: A. An example of exonic, amino-acid changing SNPs detection in esophageal cancer before (left) and after (right) filtering mouse sequences during the data analysis of whole exome sequencing of PDX tumor tissues. More than 40% of amino-acid changing SNPs detected in xenograft samples were due to mouse sequence contaminations (yellow circle on the left panel). Removal of mouse reads significantly reduced the false positive SNP calls but did not affect the capability to detect real mutations/SNPs in the human compartment. Pink circle, amino-acid changing alterations detected in human blood samples. Blue circle, amino-acid changing alterations detected in case-matched original tumor samples. Yellow circle, amino-acid changing alterations detected in derived PDX samples. The numbers and percentages present genetic alterations, as well as their percentages, in individual or overlapped populations. **B.** A case study was shown to demonstrate the consistence of amino-acid changing alterations detected by WES between the original patient tumor and its PDX at various passages. Color-coded circles present the alterations detected in patient, P2 and P5 PDX samples. The numbers represent the alterations in each or overlapped populations**.**

Supplementary Table S1 (part I). Analysis of gene expression levels between PDX and primary tumors.
Differential expression of genes in patient and PDX tissues assessed by gene expression array in case-matched 9 paired samples. Genes up-regulated in PDXs relevant to the original tumors were summarized on the sheet entitled 'UP in PDX' and genes down-regulated in PDXs were summarized on the sheet entitled 'DOWN in PDX'. The data analysis process and detailed descriptions were outlined on a separate sheet entitled 'Summary'.

1	**Item Name**	**Description**
	Probeset ID	Affymetrix PrimeView Probeset ID
	Gene Symbol	HGNC gene symbol
	Description	Gene description
	logFC (i.e., log fold change)	Log(2) of gene expression in PDXs minus Log(2) of gene expression in the original tumors. A positive logFC indicates an upregulation in PDXs and a downregulation in the original tumors. A negative logFC indicates a downregulation in PDXs and upregulation in the original tumors. (logFC = 1) suggests a 2-fold increase in PDXs when compared to the original tumors. (logFC = −1) suggests a 2-fold decrease in PDXs when compared to the original tumors
	adj.*P*.Val	Adjusted *P* value. Usually confident threshold is set as 0.05.
**2**	**Criteria of genes selection**	**Threshold**
	adj.*P*.Val	0.05
**3**	**Differential Expression Genes**	**Top Related Pathways in KEGG (www.genome.jp/kegg/pathway.html)**
	UP_in_PDX_DOWN_in_Tumor	Cell cycle, DNA replication
	DOWN_in_PDX_UP_in_Tumor	Cell adhesion molecules, Systemic lupus erythematosus, Allograft rejection, Graft-versus-host disease, Intestinal immune network for IgA production, Type I diabetes mellitus, Viral myocarditis, Autoimmune thyroid disease, Cytokine-cytokine receptor interaction

Supplementary Table S2. A list of nonsynonymous SNPs and indels identified in the exonic regions by WES in PDXs. The detailed information includes frequency, total variations detected in 56 models, specific models, chromosome numbers, locations of genes, reference (i.e., normal) and altered nucleotide bases, type of variations (i.e., SNP and indel), reference and altered amino acids, and the related information on the dbSNP (http://www.ncbi.nlm.nih.gov/SNP/) and COSMIC databases (http://cancer.sanger.ac.uk/cancergenome/projects/cosmic/; N/A, not available).

Supplementary Table S3. A summary of CNA in 42 PDX models assessed by SNP 6.0 arrays. Copy number status of each sample (sheet 1), frequencies of amplifications (A), deletions (D) and normal copy number states (N) were shown in sheet 2.
